# A Novel Synthetic Precursor of Styryl Sulfone Neuroprotective Agents Inhibits Neuroinflammatory Responses and Oxidative Stress Damage through the P38 Signaling Pathway in the Cell and Animal Model of Parkinson’s Disease

**DOI:** 10.3390/molecules26175371

**Published:** 2021-09-03

**Authors:** Ying Guo, Zhizhong Ma, Xianling Ning, Ying Chen, Chao Tian, Xiaowei Wang, Zhili Zhang, Junyi Liu

**Affiliations:** 1 Department of Chemical Biology, School of Pharmaceutical Sciences, Peking University, Beijing 100191, China; guoying@bjmu.edu.cn (Y.G.); cheny100901@pku.edu.cn (Y.C.); tianchao@bjmu.edu.cn (C.T.); xiaoweiwang@bjmu.edu.cn (X.W.); 2 Department of Integration of Chinese and Western Medicine, School of Basic Medical Sciences, Peking University, Beijing 100191, China; zhma@bjmu.edu.cn; 3 Institute of Systems Biomedicine, School of Basic Medical Sciences, Peking University, Beijing 100191, China; ningxianling@bjmu.edu.cn; 4 State Key Laboratory of Natural and Biomimetic Drugs, Peking University, Beijing 100191, China

**Keywords:** Parkinson’s disease, styryl sulfone compounds, p38 MAPK, neuroinflammatory responses, oxidative stress

## Abstract

A novel class of styryl sulfones were designed and synthesized as CAPE derivatives by our work team, which showed a multi-target neuroprotective effect, including antioxidative and anti-neuroinflammatory properties. However, the underlying mechanisms remain unclear. In the present study, the anti-Parkinson’s disease (PD) activity of 10 novel styryl sulfone compounds was screened by the cell viability test and the NO inhibition test in vitro. It was found that **4d** exhibited the highest activity against PD among them. In a MPTP-induced mouse model of PD, the biological activity of **4d** was validated through suppressing dopamine neurotoxicity, microglial activation, and astrocytes activation. With compound **4d**, we conducted the mechanistic studies about anti-inflammatory responses through inhibition of p38 phosphorylation to protect dopaminergic neurons, and antioxidant effects through promoting nuclear factor erythroid 2-related factor 2 (Nrf2). The results revealed that **4d** could significantly inhibit 1-methyl-4-phenyl-1,2,3,6-tetrahydropyridine/1-methyl-4-phenylpyridinium (MPTP/MPP^+^)-induced p38 mitogen-activated protein kinase (MAPK) activation in both in vitro and in vivo PD models, thus inhibiting the NF-κB-mediated neuroinflammation-related apoptosis pathway. Simultaneously, it could promote Nrf2 nuclear transfer, and upregulate the expression of antioxidant phase II detoxification enzymes HO-1 and GCLC, and then reduce oxidative damage.

## 1. Introduction

Parkinson’s disease (PD) is a progressive neurodegenerative disease characterized by loss of dopaminergic neurons in the substantia nigra of the midbrain and accumulation of α-synuclein protein in the form of Lewy bodies [1]. Clinically, the main symptoms of PD are motor abnormalities including bradykinesia, tremor, and muscular rigidity, and nonmotor-related dysfunctions including genitourinary problems, emotional changes, and cognitive problems [2]. Although levodopa, as a precursor to dopamine, is regarded as the golden standard of anti-PD drugs for symptomatic relief in reducing the motor symptoms, it can produce reactive oxygen through auto-oxidation, which aggravates the injury of dopaminergic neurons in patients with PD. The long-term treatment causes adverse effects such as motor fluctuations, dyskinesias, and so on [3]. 

The pathogenic mechanism of PD is comprehensive and sophisticated, involving factors such as abnormality of neural pathways, neuroinflammation, oxidative stress, apoptosis, and mitochondrial function injury [4,5,6]. Since most single-target drugs have shown unmet results in alleviating the symptoms of PD, it is necessary to further study drugs from multifactorial pathways underlying neuronal degeneration [7]. Caffeic acid phenethyl ester (CAPE), a natural compound extracted from propolis of honeybee hives, has a variety of biological activities, such as antioxidative, anti-inflammatory, and anti-atherosclerotic [8]. Previous studies have shown that CAPE has an excellent multi-target neuroprotective effect, which could significantly improve the behavioral animal models of neurodegenerative diseases through regulating several different levels in the signal pathways [9,10,11]. Therefore, as a multi-target neuroprotective agent, CAPE is a promising drug leading to further optimization. 

**Figure 1:**
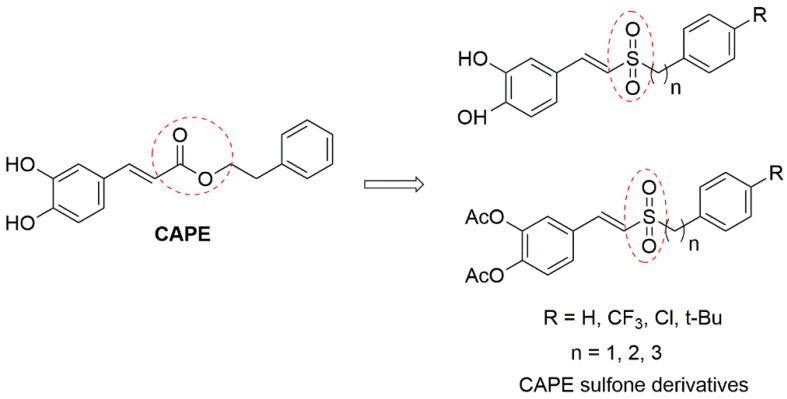
Structures of CAPE and CAPE sulfone derivatives.

Our research group introduced the sulfone group to CAPE as CAPE sulfone derivatives (Figure 1) with high metabolic stability and good blood–brain barrier permeability (disclosed for the first time in [12,13]), which exhibited greater antioxidative and anti-neuroinflammatory effects than those of CAPE in vitro models of PD. Recently, the vinyl sulfone group is often also reported in the skeletal structures of many promising neuroprotective agents, including 5-HT6 receptor antagonists [14] and caspase-6 inhibitors [15]. Besides, styryl sulfone small molecules show low toxicity and good blood–brain barrier permeability into the central nervous system (CNS) [16], which will be more efficient in the treatment of neurodegenerative disease. 

It is believed that oxidative stress and neuroinflammation are major contributors that induce the onset of PD [17]. The extensive production of reactive oxygen species (ROS) leads to lipid peroxidation and the generation of toxic products, such as nitric oxide (NO) and superoxide, which are responsible for dopaminergic neuronal loss [18]. ROS can be generated both in neurons and glia, and over-production of ROS in microglia and astrocytes elicits activation of the mitogen-activated protein kinases (MAPKs) and nuclear factor-kappa B (NF-κB) pathways. In turn, this causes increased production of pro-inflammatory mediators such as inducible nitric oxide synthase (iNOS), COX-2, the cytokines TNF-α and IL-1β, and more ROS production, creating a vicious cycle exacerbating the death of nigral neurons [19,20]. P38 MAPK could be activated upon stress events and is a central player linking oxidative stress with neuro-inflammation, and phospho-active p38 MAPK (p-p38) has been detected in the postmortem brains of PD patients and animal PD models [21]. It has been found that p38 MAPK is involved in the regulation of several transcriptional factors, including NF-κB and Nrf2 [22,23].

The neurotoxin MPTP is commonly used to generate experimental models of PD because it selectively damages the dopamine neurons, producing biochemical and neuropathological changes similar to those observed in human PD [24]. To further investigate the function of CAPE sulfone derivatives in anti-PD, 10 compounds were screened against MPTP-induced cellular and animal models of PD on the basis of those successfully synthesized before by our research group. With compound **4d** with the best anti-PD activities, we researched the inhibition of p38 phosphorylation to protect dopaminergic neurons from inflammatory responses and also antioxidant effects of promoting Nrf2 and upregulating the expression of antioxidant phase II detoxification enzymes HO-1 and GCLC. This study aimed to evaluate the protective effect of a novel class of styryl sulfone compounds in both in vitro and in vivo PD models. In addition, the selected compound was further investigated to assess its ability to regulate the PD inflammatory process and oxidative insults, and then elucidate the possible underlying mechanisms.

## 2. Results

### 2.1. In Vitro Screening against PD Activity for Styryl Sulfone Compounds 

#### 2.1.1. Evaluation of the Styryl Sulfone Compounds for Neurons’ Protection 

**Figure 2:**
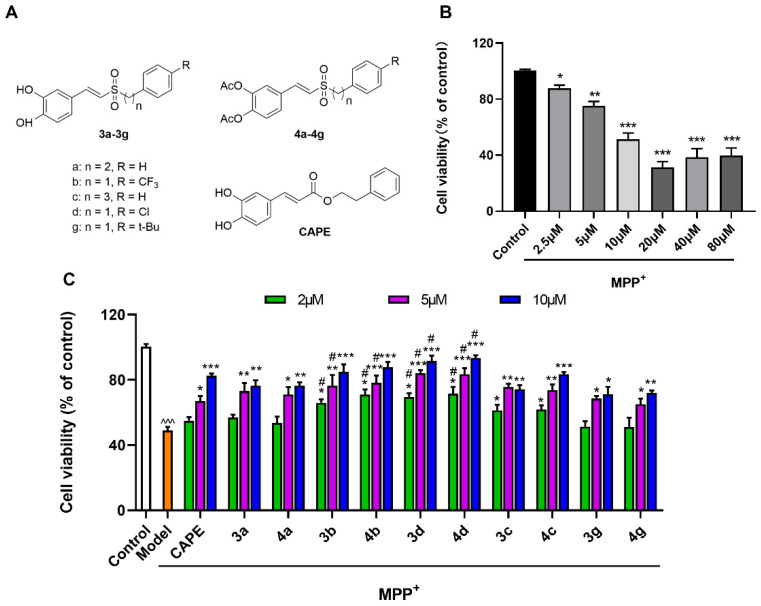
Protective effects of styryl sulfone compounds against MPP^+^-induced neurotoxicity of cultured mesencephalic neurons. (**A**) Structure of 10 styryl sulfone compounds and CAPE. (**B**) Effects of MPP^+^-induced neurotoxicity on mesencephalic neurons. Cells were treated with 2.5–80 μM of MPP^+^ for 48 h. Approximately 40–60% of cells died when they were exposed to an MPP^+^ concentration of 10 μM. (**C**) Styryl sulfone compounds block MPP^+^-induced neurotoxicity on mesencephalic neurons at 2, 5, and 10 μM. Mesencephalic neurons were pretreated by the tested compound for 3 h. Then, the cells were treated with 10 μM of MPP^+^ for 48 h. Cell viability was determined using the CCK-8 assay. The viability of untreated cells is defined as 100%. Control group: neurons were treated with phosphate-buffered saline (PBS). Model group: neurons were treated with 10 µM of MPP^+^ + PBS. Data are expressed as the mean ± SD, *n* = 3. Model group versus Control group, ^^^ *p* < 0.001; versus Model group, * *p* < 0.05, ** *p* < 0.01, *** *p* < 0.001; versus CAPE group, # *p* < 0.05.

After mesencephalic neurons were treated with different concentrations of MPP^+^ (2.5, 5, 10, 20, 40, and 80 µM), their cell viabilities decreased in a concentration-dependent manner. When mesencephalic neurons were incubated with 10 µM of MPP^+^ for 48 h, the survival rate of neuronal cells decreased by about 40–60% (Figure 2B). The inhibition of MPP^+^-induced toxicity of mesencephalic neurons by styryl sulfone compounds is shown in Figure 2. The results showed that all styryl sulfone compounds significantly display protection effects against damage induced by MPP^+^ in a dose-dependent manner. It was observed that **3b**, **3d**, **4b**, and **4d** could inhibit mesencephalic neuron injury more significantly than CAPE (Figure 2C). Among them, **4d** was the most active agent that dose-dependently blocked the MPP^+^-induced decrease of neuronal cells. Neuronal cells that received 2, 5, and 10 μM of **4d** had improved neuron viabilities by up to 71.6%, 83.5%, and 93.4% of untreated controls respectively, compared with 54.9%, 67.0%, and 82.5% in the CAPE-treated group (*p* < 0.05). 

No significant difference of inhibitory activities was observed between **3a**, **3b**, **3c**, **3d**, and **3g** and their phenolic hydroxyl derivatives **4a**, **4b**, **4c**, **4d**, and **4g.** The inhibitory effects of aromatic alkyl partially electron-absorbing substituents CF_3_- and Cl-substituted compounds (**3b**, **4b**, **3d**, and **4d**) were stronger than those of t-Bu-substituted compounds (**3g** and **4g**). The difference between aromatic alkyl-substituted compounds (*n* = 2) **3a** and **4a** and the compounds (*n* = 3) **3c** and **4c** in inhibiting the injury activity induced by MPP^+^ was not significant (Figure 2C).

#### 2.1.2. Evaluation of the Styryl Sulfone Compounds for NO Inhibition

Lipopolysaccharide (LPS) is one of the most common inflammogens used to investigate the impact of inflammation on neuron death. When microglia are activated via an inflammatory reaction induced by LPS, they can cause neuronal damage through the overproduction of proinflammatory substances, such as NO. In the previous study, our team members found that styryl sulfone compounds could significantly inhibit the release of the inflammatory mediator NO from BV-2 microglia induced by LPS [12]. 

**Figure 3:**
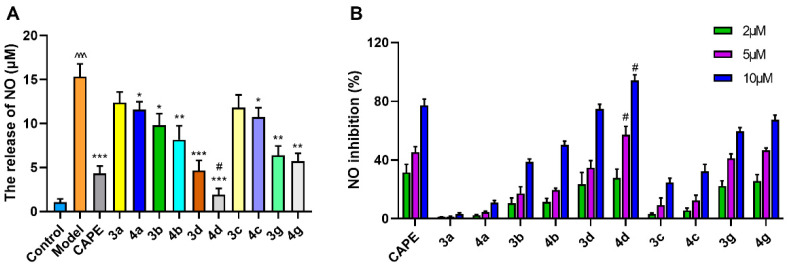
Inhibitory activities of styryl sulfone compounds against LPS-induced NO production in primary microglia culture. (**A**) Styryl sulfone compounds block LPS-induced glial release of NO at 10 μM. Control group: microglia were treated with PBS. Model group: microglia were treated with 1 μg/mL of LPS + PBS. (**B**) Inhibition effects of styryl sulfone compounds against NO production induced by LPS at 2, 5, and 10 μM. Data are expressed as the mean ± SD, *n* = 3. Model group versus Control group, ^^^ *p* < 0.001; versus Model group, * *p* < 0.05, ** *p* < 0.01, *** *p* < 0.001; versus CAPE group, # *p* < 0.05.

In primary microglia cultures, LPS caused a significant elevation of NO release from 1.05 to 15.32 μM (Figure 3A). Styryl sulfone compounds could inhibit the LPS-stimulated NO production to different degrees. Among them, **4d** showed prominent inhibitory activity against NO production in a dose-dependent manner, decreasing by about 27.8%, 57.1%, and 94.0% at 2, 5, and 10 μM respectively, compared to the Model group, and 1.3- and 1.2-fold higher activity than CAPE at doses of 5 and 10 μM (Figure 3B).

### 2.2. Validation of Anti-PD Biological Activity of ***4d*** in Animal Experiments

#### 2.2.1. **4d** Blocks MPTP-Induced Loss of Dopamine Neurons in Substantia Nigra of Mice

**Figure 4:**
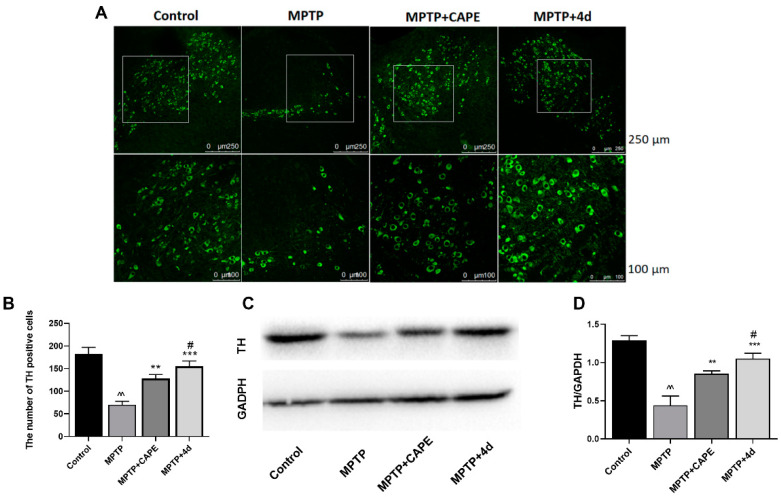
**4d** prevents loss of dopamine neurons and rescues TH expression in substantia nigra of MPTP-induced PD mice. (**A**) Representative photos showed that **4d** or CAPE at 40 mg/kg significantly protects TH-positive neurons from death induced by MPTP exposure as compared to only MPTP-exposed animals. Scale bar is 250 and 100 μm, respectively. (**B**) The number of TH-positive cells in substantia nigra for each group. (**C**) Midbrain tissues were subjected to Western blot against TH, using glyceraldehyde-3-phosphate dehydrogenase (GAPDH) as an internal control. (**D**) Densitometric analysis of changes in levels of TH, and the data were normalized against the internal control GAPDH. Data are expressed as the mean ± SD, *n* = 6. Model group versus Control group, ^^ *p* < 0.01; versus Model group, ** *p* < 0.01, *** *p* < 0.001; versus CAPE group, # *p* < 0.05.

The progressive loss of dopaminergic neurons in the substantia nigra and deletion of dopamine in the striatum are the main pathological events in PD. Tyrosine hydroxylase (TH) is the rate-limiting key enzyme in dopamine synthesis. It is commonly used as a marker for dopaminergic neurons [25]. After mice were treated with MPTP, their fluorescence intensity in the substantia nigra area significantly reduced (Figure 4A), and the number of TH-positive neurons reduced by 42.5% (Figure 4B). When mice were treated daily with 40 mg/kg of **4d** at the same time, the number of TH neurons significantly increased, ranging from 57.5% of control (MPTP treatment) to 87.1% of control (Figure 4B). Compared to CAPE, increasing the number of TH neurons to 74.2%, **4d** had a stronger inhibitory effect on the loss of dopaminergic neurons induced by MPTP (Figure 4B).

From Western blot analysis of midbrain tissue, MPTP treatment reduced the expression levels of TH in the midbrain tissue by 65.6% compared with untreated controls (Figure 4C,D). Mice that received treatment of **4d** or CAPE showed increased expression of TH protein levels, ranging from 34.4% of control (MPTP treatment) to 81.4% (**4d**) or 65.9% (CAPE) of control (Figure 4D). **4d** exhibited an optimal inhibitory effect on the decrease of mesencephalic TH expression induced by MPTP that was 1.2-fold higher than CAPE.

#### 2.2.2. **4d** Inhibits MPTP-Induced Activation of Microglia and Astrocytes in Substantia Nigra and Hippocampus of Mice

We next assessed whether **4d** could suppress neuroinflammation in MPTP-induced PD mice. Microglia and astrocytes are the major immune cells in neurodegenerative disorders such as PD. Overactivation of them is known to be involved in the continuous production of inflammatory mediators that are toxic to neurons. CD11b and glial fibrillary acidic protein (GFAP) were used as markers for microglia and astrocytes, respectively. Therefore, the effects of **4d** were tested against activation of microglia and astrocytes in substantia nigra, which help control movement and coordination to affect movement, muscle control, and balance, as well as in the hippocampus region, which is correlated with memory deficits and cognitive decline in PD [26].

**Figure 5:**
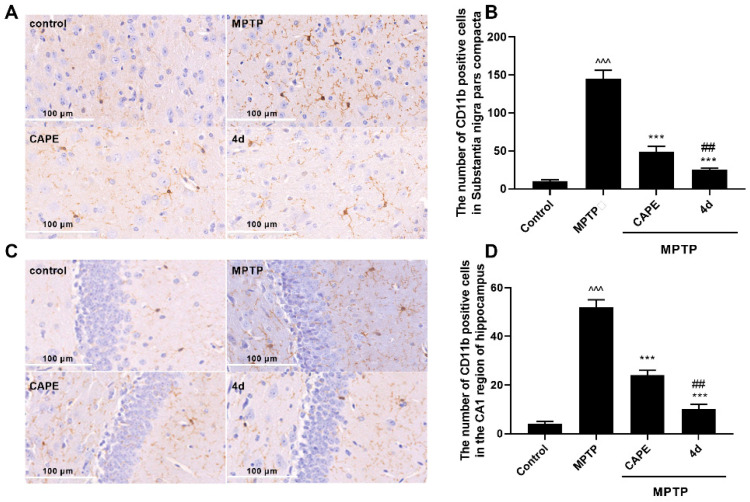
**4d** blocks activation of microglia in both the substantia nigra and hippocampus regions of Parkinson’s mice. Immunohistochemical staining was conducted against CD11b on substantia nigra sections (**A**) or hippocampus sections (**C**) and they were developed in diaminobenzidine. Scale bar: 100 μm. The number of CD11b-immunoreactivity cells was determined in substantia nigra (**B**) and in hippocampus (**D**). Data are expressed as the mean ± SD, *n* = 4. Model group versus Control group, ^^^ *p* < 0.001; versus Model group, *** *p* < 0.001; versus CAPE group, ## *p* < 0.01.

As seen in Figure 5A,C, in mice presenting MPTP-induced PD injury, microglia exhibited features of cellular hypertrophy and amoeboid morphology in both the substantia nigra and the hippocampus region. An increase in the CD11b-immuno-positive cells was evident in the substantia nigra region in the MPTP-treated mice compare to Control mice (Figure 5B). Mice administered **4d** or CAPE showed significant reductions in the number of CD11b-immuno-positive cells and activation of microglia compared with the MPTP-treated group. Figure 5C,D show that MPTP trigged the proliferation and activation of microglia in the hippocampus of mice. MPTP treatment induced an increase of CD11b-immunoreactive cells in the hippocampus region by 2.7-fold compared with the Control group, but this increase was blocked more potently by **4d** than CAPE.

**Figure 6:**
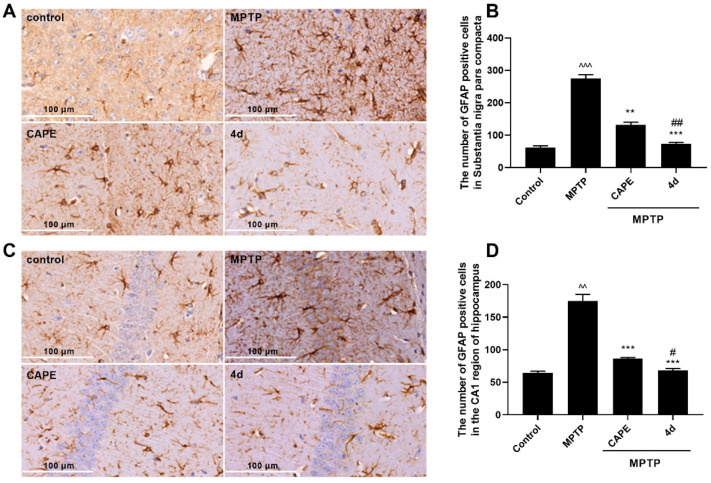
**4d** blocks activation of astrocytes in both the substantia nigra and hippocampus regions of Parkinson’s mice. Immunohistochemical staining was conducted against GFAP on substantia nigra sections (**A**) or hippocampus sections (**C**) and they were developed in diaminobenzidine. Scale bar: 100 μm. The number of GFAP-immunoreactivity cells was determined in substantia nigra (**B**) and in hippocampus (**D**). Data are expressed as the mean ± SD, *n* = 4. Model group versus Control group, ^^ *p* < 0.01, ^^^ *p* < 0.001; versus Model group, ** *p* < 0.01, *** *p* < 0.001; versus CAPE group, # *p* < 0.05, ## *p* < 0.01.

Similarly, after MPTP induction, thick and overlapping astrocytic processes indicated activation of astrocytes. The number of GFAP-positive cells in substantia nigra and in hippocampus were both significantly increased (Figure 6). Administration of **4d** obviously decreased the number of astrocytes in the substantia nigra and hippocampus regions. In contrast to CAPE, **4d** showed about 1.5- and 1.2-fold stronger inhibitory effects on activation of astrocytes in the substantia nigra and hippocampus regions respectively, and decreased the number of GFAP-positive cells close to that of the normal Control group.

### 2.3. ***4d*** Suppresses MPP^+^-Induced Inflammation by Mechanisms Associated with the p38 Signaling Pathway and Against Oxidative Stress by the Nrf2 Signaling Pathway

#### 2.3.1. **4d** Suppresses MPP^+^-Induced Activation of p38 MAPK and Downstream NF-κB Pathways in Primary Microglia

**Figure 7:**
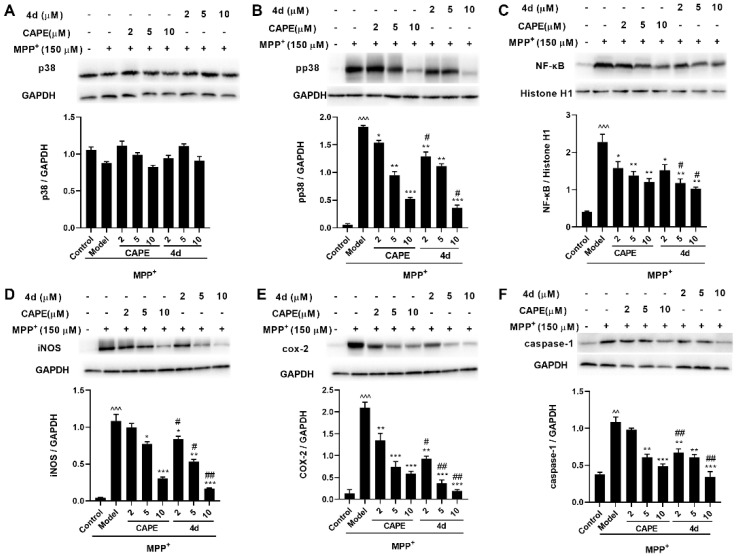
Effects of **4d** on the expression of p38, p-p38, NF-κB (in the nuclear fraction), iNOS, COX-2, and caspase-1 in MPP^+^-induced microglia. p38 (**A**), p-p38 (**B**), NF-κB (**C**), iNOS (**D**), COX-2 (**E**), and caspase-1 (**F**). Microglia were pretreated with various concentrations of **4d** or CAPE for 3 h followed by stimulation with 150 μM of MPP^+^. Then, cells were harvested after 24 h for Western blot. Densitometric analyses were performed and the data were normalized against the internal control GAPDH or Histone H1. Data are expressed as the mean ± SD, *n* = 3. Model group versus Control group, ^^ *p* < 0.01, ^^^ *p* < 0.001; versus Model group, * *p* < 0.05, ** *p* < 0.01, *** *p* < 0.001; versus CAPE group, # *p* < 0.05, ## *p* < 0.01.

P38 is the primary regulatory kinase of neuronal apoptosis induced by various stimuli [27]. Increasing evidence suggests that p38 activation is involved in the process of PD mainly through the induction of neuroinflammatory response and oxidative stress [28]. To address the mechanism of **4d** neuroprotection, we measured p-p38 levels in primary microglia induced by MPP^+^. The p-p38 levels were obviously increased in MPP^+^-treated microglia, and **4d** attenuated p38 activation in a concentration-dependent manner. Meanwhile, no changes in p38 itself were observed (Figure 7A,B). 

It has been suggested that p38 MAPK is involved in the regulation of transcription factor NF-κB. NF-κB is a transcription factor related to neuroinflammation, which exists in various types of cells in the nervous system. Typically, it is located in the cytoplasm. When the cells are stimulated by noxious stimulation, the p65 subunit of NF-κB is transferred into the nucleus to play a transcriptional role, mediating the transcriptional regulation of a variety of cytokines and inflammatory mediators [29]. Activation of p38 can further activate NF-κB nuclear transcription, and then promotes expression levels of downstream signaling proteins, iNOS, COX-2, and caspase-1, which mediate neuronal apoptosis [30]. The results revealed that the protein expressions of p-p38, NF-κB, iNOS, COX-2, and caspase-1 after MPP^+^ treatment were increased about 33.5-, 5.6-, 25.2-, 15.6-, and 2.9-fold compared to those in the Control group, whereas their increase was significantly inhibited by about 82.7%, 67.2%, 87.9%, 97.0%, and 104.4% respectively, by **4d** at 10 μM (Figure 7B–F). In contrast to CAPE, **4d** had a more robust inhibitory effect on related proteins. The protective effect of **4d** was dose-dependent as the 10 μM dose of **4d** almost completely blocked the elevation of COX-2 and caspase-1.

#### 2.3.2. **4d** Activates Nrf2 and Upregulates HO-1 and GCLC Protein Expression in MPP^+^-Induced Microglia

Oxidative stress has been implicated in the etiology of PD. It is known that Nrf2 is the main signaling pathway involved in cellular defense against oxidative stress [31]. Under normal conditions, Nrf2 is located in the cytoplasm, whereas, under conditions of oxidative stress, Nrf2 is released into the nucleus and leads to upregulation of its downstream antioxidant genes such as HO-1 and GCLC. Therefore, we examined whether **4d** could activate the Nrf2 signaling in MPP^+^-induced microglia.

**Figure 8:**
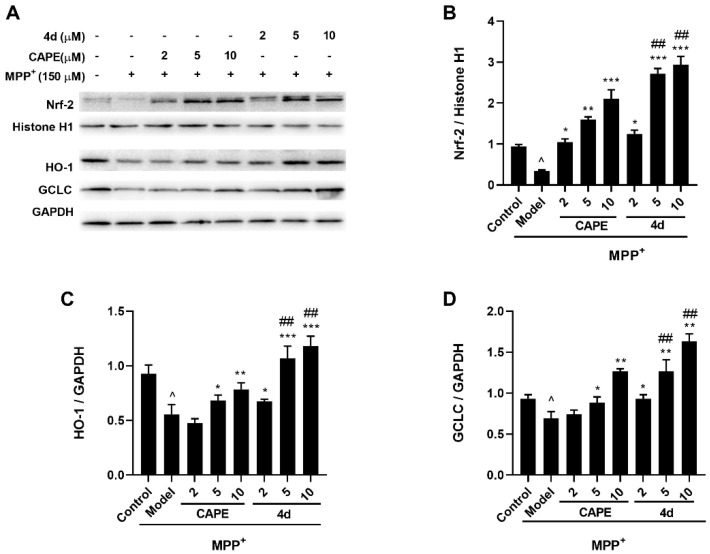
**4d** activates the Nrf2 signaling pathway in MPP^+^-induced microglia. (**A**) Microglia were pretreated with various concentrations of **4d** or CAPE for 3 h followed by stimulation with 150 μM of MPP^+^. Then, cells were harvested after 24 h and Nrf2 Western blot analysis was performed on nuclear extracts. Cytoplasmic extracts were subjected to Western blot to detect the expression of HO-1 and GCLC. Densitometric analyses were performed with the nuclear marker protein Histone H1 as an internal control for Nrf2 (**B**), and the cytosolic marker protein GAPDH as an internal control for HO-1 (**C**) and GCLC (**D**). Data are expressed as the mean ± SD, *n* = 3. Model group versus Control group, ^ *p* < 0.05; versus Model group, * *p* < 0.05, ** *p* < 0.01, *** *p* < 0.001; versus CAPE group, ## *p* < 0.01.

As shown in Figure 8, MPP^+^-treated microglia showed a significant decline of Nrf2, HO-1, and GCLC levels by 56.5%, 40.9%, and 25.8% respectively (Figure 8B–D), as compared to the Control group. Meanwhile, pretreatment with **4d** managed to promote Nrf2 nuclear accumulation in a dose-dependent manner, with a 3.5-, 7.7-, and 8.4-fold rise at 2, 5, and 10 μM respectively, as compared to the MPP^+^-treated group (Figure 8B). Additionally, **4d** elevated the HO-1 level to 100% even at 2 μM, and elevated the GCLC level to almost 100% at 5 μM (Figure 8C,D). The results suggested that **4d** could enhance the antioxidant defense ability of cells by activating the Nrf2 signaling pathway and upregulating the expression of downstream II detoxification enzymes in the MPP^+^-mediated neurotoxicity.

#### 2.3.3. **4d** Blocks Phosphorylation of p38 MAPK in an Animal Model of PD

**Figure 9:**
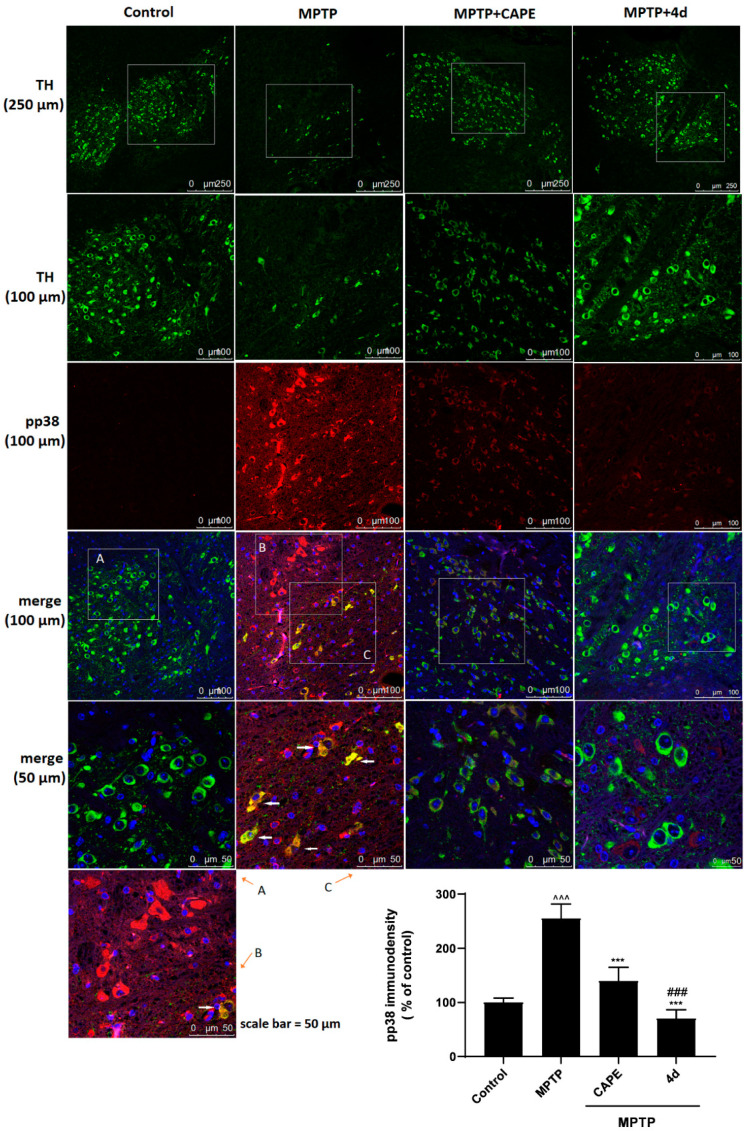
Immunostaining was conducted against TH (green) and p-p38 (red) in substantia nigral sections and the nuclei were counterstained with 4’, 6-diamidino-2-phenylindole (DAPI) (blue). There was a low level of activation of p38 (p-p38) in substantia nigral neurons of Control animals (**A**). After MPTP exposure, p-p38 (red) was obviously observed in substantia nigra of PD mice (**B**, **C**). Immunofluorescence colocalization showed the presence of p-p38 in TH (green)-positive neurons (**C**). After treatment with **4d** or CAPE (40 mg/kg) for 7 days, there was a significant decrease in p-p38 in the substantia nigra. Scale bars: 250 μm for low magnification to show the substantia nigra, 100 and 50 μm for high magnification. The bottom right corner is a quantitative statistical analysis of p-p38 immunofluorescence in the substantia nigra. Data are expressed as the mean ± SD, *n* = 4. Model group versus Control group, ^^^ *p* < 0.001; versus Model group, *** *p* < 0.001; versus CAPE group, ### *p* < 0.001.

In order to determine the molecular mechanism, we further examined whether **4d** exerts a neuroprotective effect by suppressing p38 phosphorylation activation in MPTP-induced PD mice. Immunofluorescence was used to colocalize p-p38 and TH in the substantia nigra of mice. The red immunostaining was significantly enhanced by neurotoxin MPTP, indicating activation of the p38 molecular pathway compared to the Control group (Figure 9A). Dramatically increased phosphorylation of p38 was observed (Figure 9B,C) and colocalized with TH in dopaminergic neurons (Figure 9C), which revealed that p38 phosphorylation was associated with the degeneration of dopaminergic neurons.

Conversely, levels of p-p38 protein in the substantia nigra were significantly reduced with **4d** treatment for 7 days, ranging from 255.2% of Control (MPTP treatment) to 70.3% of Control (Figure 9). Compared to CAPE, **4d** had a stronger neuroprotective action, which almost completely blocked the activation of the p38 molecular pathway.

## 3. Discussion

In our previous research, the antioxidative and anti-neuroinflammatory properties of novel styryl sulfone compounds were initially confirmed in in vitro models of PD by PC-12 cells [12]. In this study, we further examined the relationships between structure activity by using primary cultured neurons and microglial cells. On the basis of the results of our study, acetylated compounds showed more effective neuroprotective effects compared with phenolic hydroxyl compounds and CAPE. Comparison of substituted groups on the aromatic ring demonstrated that the electron-withdrawing group chloro exhibited the most potent activity. The compound with the highest activity, **4d**, was demonstrated to display promising neuroprotection against PD by suppressing activation of the p38 signaling pathway. 

Increasing evidence suggests that p38 is involved in the process of PD mainly through the induction of neuroinflammatory response and neuronal apoptosis [32]. Previous studies have shown that CAPE could regulate the p38 signaling pathway to attenuate the neuroinflammatory response in the hippocampus on LPS-induced microglial activation [9], and it could also protect neurons against glutamate-induced excitotoxicity and extend the survival of amyotrophic lateral sclerosis (ALS) mice by inhibiting phosphorylation of p38 [33,34]. However, as a neuroprotective agent, CAPE has low solubility and permeability, which reduce its bioavailability. **4d**, as a CAPE sulfone derivative with high metabolic stability and good blood–brain barrier permeability, has exhibited excellent drug-like properties. To elucidate the underlying mechanism, we focused on the study of p38 signaling, which played a crucial role in MPTP-induced microglial activation and neuronal degeneration.

Our data confirmed that **4d** was able to block MPTP/MPP^+^-induced p38 MAPK activation in both in vitro and in vivo PD models. We found that MPTP exposure could induce p38 phosphorylation in the substantia nigra of mice. Neurons in substantia nigra appeared shrunken and dead, with decreased TH immunostaining and increased p-p38 immunostaining. Additionally, increased p-p38 immunostaining was observed to colocalize within the TH-positive neurons. As expected, **4d** reversed this trend by blocking the MPTP-induced level of phosphorylated p38. Meanwhile, the activated microglia and astrocytes in the substantia nigra and hippocampus were observed in the MPTP mice, whereas the activation of microglia and astrocytes was significantly blocked by **4d**. Neuroinflammation is a type of immune response in the CNS and is observed in neurodegenerative disorders, such as PD and Alzheimer’s disease (AD). Accumulating evidence indicates that p38 is involved in the induction of neuroinflammation. Our findings suggested that **4d** may attenuate inflammatory responses associated with microglial and astrocytic activation in the substantia nigra of MPTP-induced PD model mice, leading to rescue dopaminergic neuron degeneration from MPTP toxicity via anti-inflammatory activity. It is likely that the anti-inflammatory property of **4d** is at least in part obtained via blocking p38 activation.

It is well-known that p38 regulates the transcriptional activity of NF-κB. Activation of p38 leads to the nuclear translocation of NF-κB p65 and activation of downstream signaling pathways [35]. We observed the effects of **4d** on the regulation of the p38 MAPK/NF-κB-mediated neuroinflammation-related apoptosis pathway in PD cell models. NF-κB is one of the hubs of pro-inflammatory gene expression, which plays a vital role in the regulation of iNOS and COX-2 gene transcription and protein expression. Caspase-1 is an inflammatory caspase that has multiple functions in response to inflammatory stimuli and in the regulation of cell death. The activation and high expression of iNOS and COX-2 can play a cytotoxic role through many mechanisms, inducing oxidative stress and promoting excitotoxicity of glutamate, and then activating caspase-1, to mediate the production of downstream inflammatory factors. This leads to the injury of dopamine neurons and continues to activate microglia, which continues to release inflammatory factors, expands the inflammatory response, and leads to progressive neuronal damage [36]. Our results showed that **4d** could significantly inhibit MPP^+^-induced activation of p38 in primary microglia. Consistent with the activation of p38, our research found that NF-κB activation and the levels of iNOS and COX-2 were increased in MPP^+^-induced PD cell models, as well as caspase-1 activation. Those were all effectively reversed by **4d**. The above studies suggest that **4d** can exert an anti-inflammatory role via inhibition of p38/NF-κB pathway activation.

Oxidative stress caused by excess ROS production plays a critical role in the pathogenesis of PD [5]. The Nrf2 system is the main signaling pathway involved in cellular defense against oxidative stress [37]. Activation of this pathway can induce the expression of antioxidant enzymes, such as HO-1 and GCLC, and eliminate reactive oxygen species in time to reduce the damage of dopaminergic neurons caused by parkinsonian neurotoxins, including MPTP, MPP^+^, rotenone, as well as hydrogen peroxide, in both in vitro and in vivo studies [38]. Growing evidence has shown that sustained activation of p38 leads to degradation of Nrf2, which contributes to oxidative stress and inflammatory responses [39,40]. In this study, MPP^+^ caused oxidative stress, as indicated by the downregulation of Nrf2 and Nrf2-regulated phase II detoxification enzymes, HO-1 and GCLC, in primary microglia. Treatment with **4d** induced nuclear translocation of Nrf2 and increased its downstream protein expression levels of OH and GCLC. These results suggest that the neuroprotective effect of **4d** is related to activating the Nrf2-regulated antioxidant defense pathway.

**Figure 10:**
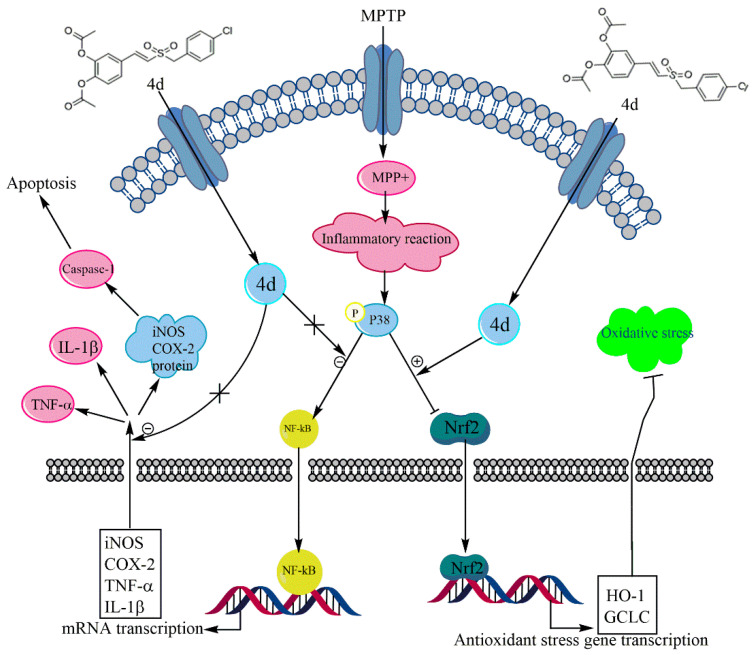
Schematic showing the protection mechanism of **4d** in the MPTP/MPP^+^-induced model of PD.

In conclusion, our results demonstrated that the new styryl sulfone neuroprotective agent, **4d**, had a significant protective effect on the PD mouse model and cell model induced by MPTP/MPP^+^. **4d** protected dopaminergic neurons from MPTP/MPP^+^-induced neurotoxicity, possibly through dual mechanisms, including anti-inflammation by inhibition of the p38 MAPK and NF-κB pathways, and antioxidation by activation of the Nrf2 pathway (Figure 10). **4d** may therefore serve as a useful therapeutic agent for the treatment of PD.

As a whole, introducing the sulfone group to CAPE leads to improvements in neuroprotective effects. The results of this study provide compelling evidence in the search for more potent multifunctional neuroprotective agents to protect against inflammatory processes and oxidative stress for treating neurodegenerative diseases, especially PD. In addition, it was reported for the first time that styryl sulfone CAPE derivatives could play an anti-PD role through the regulation of the p38 signaling pathway, which laid a foundation for the discovery of ideal effective targets for the treatment of PD.

## 4. Materials and Methods

### 4.1. Chemicals

Ten novel styryl sulfone compounds and the lead compound CAPE were obtained from Professor Jun-yi Liu, School of Pharmaceutical Sciences, Peking University. The structures were confirmed by ^1^H NMR, ^13^C NMR, and high-resolution mass spectrometry (HRMS), and the purity was determined by high-performance liquid chromatography (HPLC). The specific structures are shown in Figure 2A, and the full chemical names are listed in Supplementary Table S1.

### 4.2. Neuronal Cell Cultures and Assessment of Cell Viability

Primary cultures of mesencephalic neurons dissected from embryonic day 17 rat embryos (Sprague Dawley) were prepared as previously described [41]. Briefly, freshly dissected mesencephalons were dissociated and the cells were seeded at a density of 2 ×10^5^ cells/mL on poly-D-lysine (2 μg/mL; Sigma) pre-coated 24-well plates in Dulbecco’s Modified Eagle Medium/Nutrient Mixture F-12 (Gibco), supplemented with 2% B27 supplement (Gibco) and 10% fetal bovine serum (FBS). Cytosine arabinoside was added to the culture medium 24 h after initial plating to inhibit glial cell proliferation. Cultures were used 5 days after preparation. After cell attachment, mesencephalic neurons were preincubated by the tested compounds with the final concentrations of 2, 5, and 10 μM respectively, for 3 h, then followed by treatment with MPP^+^ (diluted with medium to a final concentration of 10 μM; Sigma D048) for 48 h. Cell viability was measured by the CCK-8 assay (Cat. No. CK04, Dojindo, Kyushu, JPN). The absorbance was detected at 450 nm. The experiment was repeated three times. The viability of cells was calculated according to the following formula:

Cell Viability (%) = (OD _Compound group_/OD _Control group_) × 100%

### 4.3. Primary Microglia Cultures and MPP^+^ Stimulation

As previously described [42], primary microglia cultures were prepared from the brain of the embryonic day 14–15 rats (Sprague Dawley) and plated on poly-D-lysine pre-coated cell-culture flasks. Cells were maintained in high-glucose Dulbecco’s Modified Eagle Medium (DMEM, Gibco, USA), containing 10% FBS, 100 U/mL penicillin, and 100 mg/mL streptomycin in a humidified atmosphere of 5% CO_2_ at 37 °C. After 14 days, microglia were selectively harvested in culture medium by shaking the flasks at 180 rpm for 1 h. Purity of microglia cultures was greater than 90%, as determined by CD11b staining. Microglia were plated in 24-well microplates at a density of 2 × 10^5^ cells/mL. Cells were preincubated for 3 h with **4d** or CAPE before addition of MPP^+^ (150 μM) for a further 24 h.

### 4.4. Determination of NO Inhibition

Primary microglia were plated in 24-well microplates at a density of 1 × 10^6^ cells/mL. After cell attachment, microglia were pre-treated with different concentrations of compounds for 3 h and then post-treated with LPS (1 µg mL^−1^ as a final concentration). The NO assay kit (Cat. No. E1030, Applygen Technologies Inc., Beijing, China) was used to carry out nitrite assays after 48 h, and the release amount of NO was determined by the Griess assay. The individual values were determined by measuring the absorbance at 540 nm and calculated according to the standard curve. The percentage of inhibition of NO was obtained from the following formula:

NO inhibition rate (%) = (NO _Model group_ − NO _Compound group_)/(NO _Model group_ − NO _Control group_) × 100%.

### 4.5. Animals and Treatment

Male C57BL/6J SPF mice (9–10 weeks, weighting 22–25 g) were purchased from Beijing Vital River Laboratory Animal Technology Co., Ltd. (Production certificate: SCXK (Beijing)). Mice were housed in a room maintained at a temperature of 22 ± 1 °C under a 12/12 h light–dark cycle, and adapted to the environment for 2 to 4 days.

The mice were randomly divided into four groups (*n* = 12), including Control group, MPTP group, MPTP + CAPE group, and MPTP + **4d** group. To make the PD model, 15 mg/kg MPTP (Sigma, M0896) dissolved in 0.9% saline was injected on the 1st day, 20 mg/kg MPTP on the 2nd day, and 30 mg/kg MPTP daily for the next 5 consecutive days. Mice were administered **4d** or CAPE by oral gavage (p.o.), suspended in saline containing 1% CMC-Na, at 40 mg/kg for seven consecutive days once only at 1 h prior to MPTP injection. The mice in the Control group received injections of 0.9% saline once a day for seven consecutive days. Animals were killed two weeks after the first injection.

### 4.6. Immunohistochemistry

Animals (*n* = 6 per group) were anesthetized with 5% chloral hydrate solution 2 weeks after the first MPTP injection and perfused transcardially with phosphate-buffered saline (PBS) followed by paraformaldehyde (4%, *w*/*v*) in PBS. Sequential coronal sections (5–7 μm thick) were acquired from paraffin-embedded tissue containing substantia nigra (bregma −2.54 to −3.40 mm) and hippocampus (bregma −1.58 to −2.80 mm). Paraffin sections were prepared by the Department of Pathology, School of Basic Medical Sciences, Peking University. 

The immunohistochemical staining of CD11b and GFAP was performed with the rabbit two-step detection kit (Cat. No. PV-9001, Beijing Zhongshan JinQiao Biotechnology Co., Ltd., China) and goat two-step detection kit (Cat. No. PV-9003, Beijing Zhongshan JinQiao Biotechnology Co., Ltd., China). In brief, tissue sections were incubated successively with 3% hydrogen peroxide (H_2_O_2_), rabbit anti-CD11b, or goat anti-GFAP (Supplementary Table S2 for antibody information). Then, they were incubated with horseradish-peroxidase-conjugated anti-second antibody, visualized with 3,3’-diaminobenzidine (DAB), then dyed and sealed with neutral resin. To adequately quantify immuno-positive cells, we used the digital slide scanning system (Nanozoomer Digital Pathology, NDP) to capture images and counted immuno-positive cells by Leica Q Color (G) image analysis software.

### 4.7. Immunofluorescence Assay

Paraffin sections were dewaxed in xylene and rehydrated in graded alcohol and PBS (pH 7.4), then processed for immunofluorescence staining. For double-immunostaining, TH and p-p38 sections were simultaneously incubated with antibodies against TH and p-p38 antibodies overnight at 4 °C, and dylight 488-labeled goat anti-chicken IgY was used to detect TH-positive cells and dylight 550-labeled goat anti-mouse IgG was used to detect p-p38-expressing cells. Then, the nuclei were counterstained with DAPI. They were viewed under a confocal microscope (TCS-SP5, Carl Zeiss Microscopy GmbH, Jena, Germany). The immuno-density value of p-p38 was measured by Imagepro-plus software (Media Cybernetics, Silver Spring, CA, USA) and TH-positive neurons were detected by Leica Q Color (G) image analysis software.

### 4.8. Immuno-Positive Cell Counting

The number of immuno-positive cells was counted by two observers in a double-blind manner. After defining the boundary of the substantia nigra and hippocampus at low magnification (×10 objective), immunoreactive cells in the section were counted at a higher magnification (×40 objective). In immunofluorescence experiments, immuno-positive cells were counted only when their nuclei were optically visible for avoiding double counting. The total number of TH^+^ neurons was determined by the stereological method [43,44,45]. Cell counting was performed on one side of the brain. Six to eight evenly spaced brain slices from each animal were immunoreacted. A mean value for the number of immuno-positive cells was deduced by averaging the counts of 3–6 animals in each different group.

### 4.9. Protein Extraction and Western Blot Analysis

Western blot analysis was performed on midbrain extracts, cell cytoplasmic, and nuclear extracts. Midbrain tissues were dissected and lysed with ultrasound for no more than 30 s on ice in RIPA buffer (50 mM Tris (pH 7.4), 150 mM NaCl, 1% Triton X-100, 1% sodium deoxycholate, 0.1% SDS, sodium orthovanadate, sodium fluoride, EDTA) containing protease inhibitor cocktail (Cat. No. P1266, Applygen Technologies Inc., Beijing, China) and 1 mM of phenylmethanesulfonyl fluoride (PMSF), and incubated at 4 °C for 30 min. After centrifugation at 12,000× *g* at 4 °C for 15 min, the supernatant (containing the total protein fraction) was collected. 

The extraction of cytoplasmic and nuclear protein from primary microglia was performed by the Nuclear and Cytoplasmic Protein Extraction Kit (Cat. No. P0027, Beyotime, Shanghai, China), according to the manufacturer’s instructions. Briefly, cells were collected and resuspended in cytoplasmic protein extraction solution A containing 1 mM of PMSF and protease inhibitor cocktail. After a 5 s vortex, the tubes were incubated for 20 min on ice to promote lysis. Then, the cytoplasmic protein extraction solution B was added, and vortexed for 5 s. Next, the sample was centrifuged at 12,000× *g* for 15 min at 4 °C. The supernatant containing the cytosolic fraction was immediately frozen for further analysis. The pellet was resuspended in nuclear protein extraction solution supplemented with 1 mM of PMSF. After 5 vortexes for 40 min on ice and centrifuging at 12,000× *g* for 15 min at 4 °C, the supernatants containing the nuclear extracts were obtained.

Protein concentration was determined by the BCA protein assay kit (Thermo Scientific, Waltham, MA, USA), where 10–50 µg protein samples were loaded onto 10% or 12% SDS-polyacrylamide gel. After gel electrophoresis, proteins were transferred onto polyvinylidene difluoride (PVDF) membranes. Membranes were blocked with 5% skimmed milk at room temperature for 1 h, then incubated overnight at 4 °C with antibodies against TH, p38, p-p38, iNOS, COX-2, caspase-1, NF-κB, HO-1, GCLC, or Nrf2, respectively (details of the antibodies are shown in Supplementary Table S2). After incubation with horseradish-peroxidase-conjugated secondary antibodies at room temperature for 1.5 h, ECL luminescent reagent (cat. No. 32109, Millipore, Burlington, MA, USA) was added and the bands of proteins were observed using the ChemiDoc XRS system (Bio-Rad Laboratories, Inc., CA, USA) and analyzed by Image Lab Version 3.0. Blots were normalized with GAPDH or Histone H1 as appropriate.

### 4.10. Statistical Analysis

Data were expressed as mean ± SD of at least three samples prepared separately from different groups of animals, and all experiments in vitro were performed three times. Statistical analysis of the data between multiple groups was performed using one-way ANOVA followed by Dunnett’s or Bonferroni’s multiple comparisons post-test. Paired *t*-tests were used when two groups were compared. The analyses were performed with the statistical software GraphPad Prism 8.0 (GraphPad Software Inc., San Diego, CA, USA). A value of *p* < 0.05 was considered statistically significant.

## Data Availability

Data supporting reported results may be supplied upon request by authors.

## Supplementary Materials

The following are available online, Table S1: The structure and chemical name of styryl sulfone compounds, Table S2: The antibody information.

## Sample Availability

Samples of the compounds (ten novel styryl sulfone compounds and the lead compound CAPE) are available from the authors.
