# Supplementary material for: A Novel Synthetic Precursor of Styryl Sulfone Neuroprotective Agents Inhibits Neuroinflammatory Responses and Oxidative Stress Damage through the P38 Signaling Pathway in the Cell and Animal Model of Parkinson’s Disease
Source: Molecules. 2021 Sep 3;26(17):5371. doi: 10.3390/molecules26175371 (PMC8433997; doi:10.3390/molecules26175371)
Supplement: Supplementary file 1 [file molecules-26-05371-s001.zip › molecules-1305752-supplementary.pdf]

Supplementary Material Table.

Table S1. The structure and chemical name of styryl sulfone compounds

Compound	Structure diagram	Chemical names
CAPE	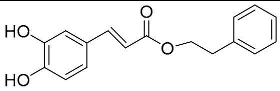	3,4-Dihydroxy-trans-cinnamic acid phenethyl ester
**3a**	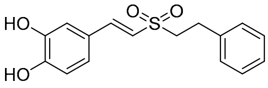	(*E*)-4-[2-((2-phenylethyl)sulfonyl)vinyl]benzene-1,2-diol
**3b**	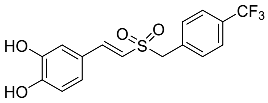	(*E*)-4-[2-((4-trifluoromethyl)benzyl)sulfonyl)vinyl]benzene-1,2-diol
**3c**	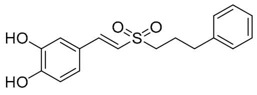	(*E*)-4-[2-((3-phenylpropyl)sulfonyl)vinyl]benzene-1,2-diol
**3d**	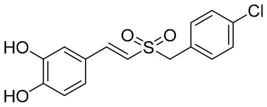	(*E*)-4-[2-((4-chlorobenzyl)sulfonyl)vinyl]benzene-1,2-diol
**3g**	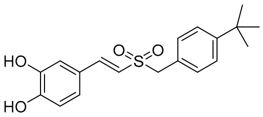	(*E*)-4-[2-((4-(tertbutyl)benzyl)sulfonyl)vinyl]benzene-1,2-diol
**4a**	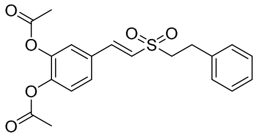	(*E*)-acetic acid 2-acetoxy-4-[2-(2-phenylethanesulfonyl)vinyl]phenyl ester
**4b**	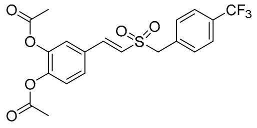	(*E*)-acetic acid 2-acetoxy-4-[2-(4-trifluoromethylphenylmethanesulfonyl)vinyl]phenyl ester
**4c**	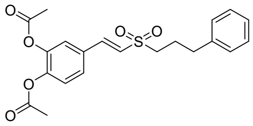	(*E*)-acetic acid 2-acetoxy-4-[2-(3-phenylpropanesulfonyl)vinyl] phenyl ester
**4d**	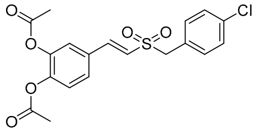	(*E*)-acetic acid 2-acetoxy-4-[2-(4-chlorophenylmethanesulfonyl)vinyl] phenyl ester
**4g**	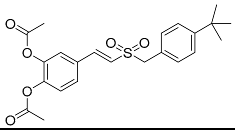	(*E*)-acetic acid 2-acetoxy-4-[2-(4-tertbutylphenylmethanesulfonyl) vinyl] phenyl ester

Table S2. The antibody information

Antibody name	Dilution ratio.	Reagent manufacture	Cat. no.
Tyrosine hydroxylase (TH) chicken polyclonal antibody	1:500	Abcam, San Francisco, CA, USA	ab76442
Dylight 488 labeled goat anti-chicken IgY	1:500	Thermo Fisher Scientific, Waltham, MA, USA	SA5-10070
Dylight 550 labeled goat anti-	1:500	Thermo Fisher Scientific,	SA5-

mouse IgG		Waltham, MA, USA	10173
Dylight 650 labeled donkey anti-rabbit IgG	1:500	Thermo Fisher Scientific, Waltham, MA, USA	SA5-10041
CD11b rabbit monoclonal antibody	1:1000	Abcam, Cambridge, MA, USA	ab133357
GFAP goat polymonoclonal antibody	1:1000	Abcam, Cambridge, MA, USA	ab53554
p38 rabbit monoclonal antibody	1:1000	Abcam, Cambridge, MA, USA	ab170099
p-p38 MAPK mouse monoclonal antibody	1:200	Santa Cruz Biotechnology, Dallas, USA	sc-166182
iNOS rabbit monoclonal antibody	1:1000	Abcam, Cambridge, MA, USA	ab178945
COX-2 rabbit monoclonal antibody	1:1000	Cell Signaling Technology, Inc., MA, USA	12282T
Caspase-1 rabbit polyclonal antibody	1:1000	Millipore, Burlington, MA, USA	AB1871
NF-κB p65 mouse monoclonal antibody	1:1000	Santa Cruz Biotechnology, Dallas, USA	sc8008
HO-1 rabbit monoclonal antibody	1:1000	Abcam, Cambridge, MA, USA	ab68477
GCLC rabbit monoclonal antibody	1:1000	Abcam, Cambridge, MA, USA	ab190685
Nrf2 rabbit polyclonal antibody	1:1000	Abcam, Cambridge, MA, USA	ab137550
Monoclonal antibody to GAPDH mouse	1:1000	R&D Systems, Minneapolis, MN, USA	MAB5718
Histone H1 mouse monoclonal antibody	1:1000	Santa Cruz Biotechnology, Dallas, USA	sc-8030
Horseradish enzyme labeled goat anti-mouse IgG	1:1000	Beijing Zhongshan Jinqiao Biotechnology, Beijing, China	ZB-5305
Horseradish enzyme labeled goat anti-rabbit IgG	1:5000	Beijing Zhongshan Jinqiao Biotechnology, Beijing, China	ZB-2301
Horseradish enzyme labeled rabbit anti-goat IgG	1:5000	Beijing Zhongshan Jinqiao Biotechnology, Beijing, China	ZB-2306
